# Pleural Effusion in Spinal Deformity Correction Surgery- A Report of 28 Cases in a Single Center

**DOI:** 10.1371/journal.pone.0154964

**Published:** 2016-05-11

**Authors:** Weiqiang Liang, Bin Yu, Yipeng Wang, Guixing Qiu, Jianxiong Shen, Jianguo Zhang, Hong Zhao, Yu Zhao, Ye Tian, Shugang Li

**Affiliations:** Department of Orthopedics, Peking Union Medical College Hospital, Chinese Academy of Medical Sciences & Peking Union Medical College, Beijing, P.R. China; City of Hope, UNITED STATES

## Abstract

**Objectives:**

To analyze the occurrence, risk factors, treatment and prognosis of postoperative pleural effusion after spinal deformity correction surgery.

**Methods:**

The clinical and imaging data of 3325 patients undergoing spinal deformity correction were collected from the database of our hospital. We analyzed the therapeutic process of the 28 patients who had postoperative pleural effusion, and we identified the potential risk factors using logistic regression.

**Results:**

Among the 28 patients with postoperative pleural effusion, 24 (85.7%) suffered from hemothorax, 2 (7.1%) from chylothorax, and 2 (7.1%) from subarachnoid-pleural fistula. The pleural effusion occurred on the convex side in 19 patients (67.9%), on the concave side in 4 patients (14.3%), and on both sides in 4 patients (14.3%). One patient with left hemothorax was diagnosed with kyphosis. The treatment included conservative clinical observation for 5 patients and chest tube drainage for 23 patients. One patient also underwent thoracic duct ligation and pleurodesis. All of these treatments were successful. Logistic regression analysis showed that adult patients(≥18 years old), congenital scoliosis, osteotomy and thoracoplasty were risk factors for postoperative pleural effusion in spinal deformity correction surgery.

**Conclusions:**

The incidence of postoperative pleural effusion in spinal deformity correction surgery was approximately 0.84% (28/3325), and hemothorax was the most common type. Chest tube drainage treatment was usually successful, and the prognosis was good. Adult patients(≥18 years old), congenital scoliosis, and had undergone osteotomy or surgery with thoracoplasty were more likely to suffer from postoperative pleural effusion.

## Introduction

Spinal deformities (scoliosis or kyphosis) are not rare in orthopedics. Some patients can be treated conservatively, while for others, spinal deformity correction surgeries are necessary. Spinal deformity correction surgery is an effective treatment, however, it is a technique demanding procedure that is associated with some risks. Complications can occur during or after the operation[1–6]. Pleural effusion can also occur after spinal deformity correction surgery, especially in patients with a thoracic curve that needs to be corrected[7,8]. However, to our knowledge, few reports in the literature focus on this complication[9–11].

The purpose of this report is to analyze the occurrence of pleural effusion after spinal deformity correction surgery at a single institution and to determine the prognosis of and possible risk factors for this complication.

## Materials and Methods

### Demographic data

Approval was obtained from the Institutional Review Board (IRB) of Peking Union Medical College Hospital before the study. Since this was a retrospective study and the information of the patients was anonymized, no written informed consent was obtained from the participants. We then retrospectively reviewed the medical records and radiographic data of the patients who underwent correction surgery at our hospital from January 2003 to December, 2014. The patients who underwent wound debridement only, implant removal only or growing rod procedure were excluded. There were a total of 3325 patients, 1123 males and 2202 females, with an average age of 16.8 years old (range, 2 to 70). Of these patients, 820 were adults (≥18 years old) and 2505 were younger than 18 years. The etiologies of the 3325 patients were listed in Table 1.

**Table 1:** Etiology of the patients.

Pleural effusion	IS(infantile and juvenile)	AIS	IS(adult)	congenital scoliosis	NF	NMS	other scoliosis	kyphosis	Total
Yes	0	2	5	18	2	0	0	1	28
No	14	1016	395	1164	102	242	120	244	3297
Total	14	1018	400	1182	104	242	120	245	3325

All of the patients were treated with spinal deformity correction surgery under general anesthesia. The correction surgeries included anterior correction in 98 cases, posterior only surgery in 3154 cases and an anterior and posterior approach in 73 cases. Thoracoplasty was also performed in 97 patients. Nine hundred and forty-eight patients underwent an osteotomy procedure, which included posterior vertebral column resection in 117 cases, Smith-Peterson osteotomy in 183 cases, pedicle subtraction osteotomy in 194 cases, and hemivertebra resection in 454 cases. Sixty-nine cases were revision surgeries.

All of the cases were reviewed, and pleural effusion was confirmed with medical records and chest X-ray films. Fluid was obtained via pleurocentesis or thoracic tube drainage, and its characteristics were determined.

### Statistics

SPSS 17.0 for Windows (SPSS, Inc., Chicago, IL) was used for the statistical analysis. Differences in patient characteristics between different groups were compared using chi-squared or Fisher’s exact tests for categorical variables. Logistic regression models were used to determine the risk factors for pleural effusion. Odds ratios (ORs) and 95% confidence intervals (95% CIs) for pleural effusion were reported. All tests were 2-sided and were considered statistically significant if the P value was less than 0.05.

## Results

The curve types among the 3080 scoliosis patients were as follows: 2 cervicothoracic curve cases(Lenke classification not applicable), 915 single thoracic curve cases(Lenke classification type 1 or alike), 552 thoracolumbar/lumbar curve cases(Lenke classification type 5 or alike), 196 double thoracic curve cases(Lenke classification type 2 or alike), 657 thoracic(major curve) and lumbar curve cases(Lenke classification type 3 or alike), 512 thoracic and lumbar curve(major curve) cases(Lenke classification type 6 or alike), 1 cervical and thoracic curve and 1 cervicothoracic curve and thoracic curve each case(Lenke classification not applicable), 244 triple curve cases(Lenke classification type 4 or alike). The other 245 cases were kyphosis patients. The mean preoperative coronal Cobb angle of the major curve in scoliosis patients was 60.3 degree (range, 18~165), which was corrected to 22.4 degree (range, 0~125), with an average correction rate of 67.4% (range, 0~100%) and the mean fusion range was 9.9 vertebrae (range, 2~19). The mean preoperative Cobb angle of the kyphosis was 69.7 degree (range, 26~137), which was corrected to 31.5 degree (range, 0~102), with an average correction rate of 55.8% (range, 0~100%) and the mean fusion range was 7.1 vertebrae (range, 2~15).

Twenty-eight patients suffered from pleural effusion after spinal deformity correction surgery, 9 cases were males, and 19 were females. The curve types among the 27 scoliosis patients with pleural effusion were as follows: 15 single thoracic curve cases(Lenke classification type 1 or alike), 3 double thoracic curve cases(Lenke classification type 2 or alike), 6 thoracic(major curve) and lumbar curve cases(Lenke classification type 3 or alike), 1 thoracic and lumbar curve(lumbar major, both structural) case(Lenke classification type 6 or alike), and 2 triple curve(major thoracic curve) cases(Lenke classification type 4 or alike). The other case was kyphosis patient. The mean preoperative coronal Cobb angle of the major curve in these 27 scoliosis patients was 76.4 degree (range, 35~125), which was corrected to 38.3 degree (range, 1~86), with an average correction rate of 50.4% (range, 0~97%) and the mean fusion range was 11.6 vertebrae (range, 5~16). The preoperative Cobb angle of the kyphosis patient was 50 degree, after operation, the kyphosis was corrected to 28 degree, with a correction rate of 44% and the fusion range was 10 vertebrae. The surgical procedures included anterior correction in 3 cases, posterior-only surgeries in 24 cases and anterior and posterior correction in 1 case. The details of the patients with pleural effusion were listed in Table 2.

**Table 2:** Details of the patients with pleural effusion.

Case number	etiology	surgical approach	osteotomy	thoracoplasty	effusion nature	effusion location	treatment
1,4,6,7,24	CS	P	HV resection	no	hemothorax	convex	chest tube drainage
18	CS	P	HV resection	no	hemothorax	convex	observation
21	CS	P	HV resection	no	hemothorax	both	chest tube drainage
26	CS	P	HV resection	no	subarachnoid-pleural fistula	convex	chest tube drainage
23	CS	P	VCR	no	hemothorax	convex	observation
8	CS	P	VCR	yes	hemothorax	convex	chest tube drainage
9	CS	P	VCR	no	hemothorax	concave	chest tube drainage
10	CS	P	PSO	no	hemothorax	convex	chest tube drainage
2	CS	P		yes	hemothorax	both	chest tube drainage
3	CS	P		yes	hemothorax	convex	chest tube drainage
15	CS	P		yes	hemothorax	convex	observation
22	CS	P		no	hemothorax	both	chest tube drainage
27	CS	P		no	hemothorax	concave	chest tube drainage
28	CS	A+P		no	chylothorax	both	low fat diet and chest tube drainage
13	AIS	A		no	chylothorax	convex	low fat diet and chest tube drainage
14	AIS	A		no	hemothorax	convex	observation
5	IS(adult)	P		no	hemothorax	concave	chest tube drainage
16	IS(adult)	P	SPO	no	hemothorax	convex	chest tube drainage
17	IS(adult)	P	VCR	no	subarachnoid-pleural fistula	convex	chest tube drainage
19	IS(adult)	P	SPO	no	hemothorax	concave	chest tube drainage
25	IS(adult)	P		yes	hemothorax	convex	chest tube drainage
11	NF	A		no	hemothorax	convex	chest tube drainage
20	NF	P	VCR	no	hemothorax	convex	chest tube drainage
12	kyphosis	P		no	hemothorax	left side	observation

The results of the chi-squared test for differences between those with and without pleural effusion were listed in Table 3.

**Table 3:** Comparison between those with and without pleural effusion.

	Total cases	Cases with pleural effusion	Cases without pleural effusion	P value
Gender				
Male	1123	9	1114	0.855
Female	2202	19	2183	
Age				
<18 years	2505	16	2489	0.025
≥18 years	820	12	808	
Etiology				
CS	1182	18	1164	0.001
Non-CS	2143	10	2133	
Osteotomy				
yes	948	16	932	0.001
no	2377	12	2365	
Thoracoplasty				
yes	97	5	92	0.001
no	3228	23	3205	
Revision surgery				
yes	69	1	68	1.000
no	3256	27	3229	
Surgical approach				
anterior	98	3	95	0.142
posterior	3154	24	3130	
A+P	73	1	72	

The effusion was located on the convex side in 19 cases, on the concave side in 4 cases, and on both sides in 4 cases. One patient with left-side hemothorax was diagnosed with kyphosis. The treatment of the 28 patients included observation in 5 cases and chest tube drainage in 23 cases. The 2 patients with chylothorax were also treated with a low-fat diet. For the patients with bilateral thoracic effusion, the tubes were placed in the side with more fluid. The last case was somewhat complicated. The patient was undergoing revision surgery for postoperative paraplegia 1 year after the primary surgery. The revision surgical procedure included right-side anterior release and posterior decompression and spinal fusion. During the anterior surgery, the surgeon found that the thoracic vertebra was eggshell-like, the cortical bone was very thin, and the amount of cancellous bone was greatly reduced. The surgeon was concerned about spinal instability after the spinal release, so the anterior release was cancelled, and posterior spinal fusion and decompression was performed. After the operation, there was a large amount of thoracic drainage, and radioisotope scanning showed bilateral chylothorax that was more severe on the right side. One week later, anterior ligation of the thoracic duct was performed. However, the result was not successful, and pleurodesis was performed six weeks later. After that, there was less thoracic drainage, and the drain was successfully removed one week later.

The average duration of the chest tube drainage was 4.5 days (range, 2 to 9 days; last case not included). The average amount of drainage was 1013 ml (range, 150 to 2250 ml; last case not included), and 984 ml (range, 150 to 2250 ml) for the hemothorax cases. The last patient suffered from chylothorax after anterior and posterior surgery, and the chest tube drainage lasted for 56 days and had a total volume of 100890 ml. The overall results were all successful, and all of the patients had a good prognosis.

Multivariate logistic regression analysis showed that the risks of postoperative pleural effusion included adult patients, congenital scoliosis, and treatment with osteotomy or thoracoplasty (Table 4).

**Table 4:** Logistic regression analysis.

effusion	B	S.E	Wald	df	P value	Exp(B)	Exp(B) 95% confidence interval	
							Lower limit	Upper limit
age	0.981	0.422	5.402	1	0.020	2.668	1.166	6.105
CS	1.095	0.438	6.243	1	0.012	2.988	1.266	7.054
thoracoplasty	1.953	0.544	12.865	1	0.000	7.050	2.425	20.494
osteotomy	1.023	0.412	6.159	1	0.013	2.783	1.240	6.244

## Discussion

The surgical treatment of spinal deformity has a long history. At first, only fusion cases were selected for surgery. Later, the invention of Harrington instrumentation led the spine surgeon into a new era of spinal deformity correction surgery. Currently, correction results are more favorable and surgeries are much safer than before. However, perioperative complications are still inevitable[1–8].

Pleural effusion is a possible complication of spinal deformity correction surgery that is not well reported in the literature. Some doctors have reported that postoperative hemothorax was quite rare and was often related to misplaced pedicle screws or thoracoplasty[12–20]. All the patients with pleural effusion in the current study had surgical procedure on the thoracic spine. And between the curve type matched groups, the patients with/without pleural effusion had similar correction rate of the major curve and similar fusion range(both P>0.05).

In 2014, Sarwahi et al reported a study that focused on the misplacement of pedicle screws. In their series, a total of 40 high-risk screws were found in 25 patients. Of these 40 screws, 1 was near the pleura[16]. Ogura et al reported a massive postoperative hemothorax related to thoracic pedicle screw placement[10]. Actually, a fracture of the thoracic pedicle or pleural irritation by a longer pedicle screw can also cause post-operative hemothorax. During an osteotomy procedure, especially three-column osteotomy, pleural irritation or injury or vascular damage can be the origin of hemothorax[21–23]. Pang et al reported a case of an adolescent idiopathic scoliosis patient with massive hemothorax caused by a Gelpi retractor during posterior correction surgery, which is very rare[11]. In the current study, 24 patients suffered from hemothorax, in 2 patients, this was thought to be related to the pedicle screw insertion procedure. Correct insertion of the thoracic pedicle screws can reduce the risk of pleural effusion.

Thoracoplasty is useful for reducing the rib hump, however, it carries the risk of pleural irritation, pleural tear or intercostal vessel injury during the rib dissection and partial resection[17–20]. Min et al reported 21 cases of thoracic idiopathic scoliosis treated with thoracoplasty and pedicle screw correction. In their series, 2 patients (10%) suffered from pleural effusion, and the cause was thought to be pleural irritation from the thoracoplasty. The authors suggested leaving the rib periosteum intact to protect the pleura[17]. In Suk et al’s report, 3 cases of hemothorax were found in the 50 AIS patients who underwent thoracoplasty[19]. Yang et al performed a study comparing conventional thoracoplasty with short apical rib resection thoracoplasty (SARRT). In their series, pleural rupture during thoracoplasty occurred in 8 patients, 22 patients in the conventional thoracoplasty group and 13 patients in the SARRT group suffered from pleural effusion after surgery, and 15 and 10 patients underwent chest tube insertion, respectively[20]. Lai reported that one scoliosis patient suffered from pleural effusion 14 days after posterior pedicle screw instrumentation correction combined with thoracoplasty, he thought that this delayed pleural effusion might have resulted from pleural irritation by the fractured rib ends[18]. In the current study, of the 24 patients with hemothorax, 5 underwent thoracoplasty, and 1 developed a pleural rupture during the thoracoplasty procedure. So, when thoracoplasty procedure is performed, one should be careful when dissect and partial remove the rib, and Min et al’s suggestion of leaving the rib periosteum intact to protect the pleura is useful to avoid pleural effusion.

Chylothorax can also occur as a type of pleural effusion after spinal deformity correction surgery, although it is rarer than hemothorax. Chylothorax can occur as a result of direct injury to the thoracic duct during spinal surgery. According to Nakai et al’s report, the Harrington distraction procedure and central venous catheter insertion can also cause this complication[9]. Rames et al reported that curette penetration into the thorax, rotation and distraction maneuvers can cause chylothorax through direct injury of the thoracic duct or its tributaries[24]. Although the amount of drainage in chylothorax cases is usually considerable, conservative treatment is often successful. If this fails, surgical ligation of the thoracic duct should be considered[9,24]. In our series, one patient suffered from chylothorax after video-assisted thoracoscopic spinal fusion, and she was treated with a low-fat diet and chest tube drainage with satisfactory results[25]. In Gorham-Stout syndrome patients, osteolysis of the thoracic vertebra can also lead to chylothorax[26–28]. In our last patient with pleural effusion, Gorham-Stout syndrome was suspected, and the treatment process was not smooth. Surgical ligation of the thoracic duct was performed but failed. However, a pleurodesis procedure was successful for this patient.

In rare conditions, subarachnoid-pleural fistula can be the cause of pleural effusion. If a dural tear and a pleural tear both occur in the same patient, a subarachnoid-pleural fistula can occur and result in pleural effusion[29–32]. Pollack et al suggested surgical repair of the fistula[29], while D’Souza et al reported that management should depend on the amount of effusion, the degree of dural disruption and the progression of clinical symptoms[31]. In the current study, 2 patients suffered from this complication. The surgical procedures included 1 vertebral column resection and correction and 1 hemivertebra resection and correction. We thought that the pleural effusion might have been caused by a dural tear and pleural tear during the osteotomy. However, chest tube drainage was successful in both patients.

Regarding the risk factors for pleural effusion in spinal deformity surgery, to our knowledge, there are no related reports in the literature. However, in some reports, patients with thoracoplasty were more likely to experience this complication[17–20]. In our series, multivariate logistic regression analysis showed that thoracoplasty was a risk factor for postoperative pleural effusion. This result was consistent with the aforementioned reports. In addition, the multivariate logistic regression analysis in our series found that adult patiens, congenital scoliosis, and osteotomy may be risk factors for postoperative pleural effusion. In Grossfeld et al’s report of anterior spinal fusion surgery in children, pleural effusion was defined as a minor complication and their results showed that age >14 years was a risk factor for minor complications[33]. Shaw et al performed a study of complications in adult scoliosis surgery and determined that increasing age was associated with higher rates of major short-term complications[34]. Our result were similar to these 2 reports. Adult patients(≥18 years old) had a greater risk of pleural effusion.

Congenital scoliosis (CS) is a spinal deformity characterized by the failure of vertebra formation or segmentation. CS patients have an abnormal spinal anatomy, thus, they are prone to pleural injury during spinal surgery. Furthermore, for CS patients, especially those with a hemivertebra or a sharp Cobb angle, hemivertebra resection or osteotomy is usually necessary. In cases of severe or stiff spinal deformities, some doctors also perform osteotomy to achieve a better correction of the deformity. When performing osteotomy, especially three-column osteotomy, there is a risk of rupturing or irritating the pleura and thus increasing the risk of pleural effusion[21–23]. In Lenke et al’s retrospective multicenter review, vertebral column resections were performed in 147 patients with severe pediatric spinal deformities, and 7 patients developed pleural effusion[21]. In the current study, among the 28 patients with pleural effusion, 18 had congenital scoliosis, and the occurrence of pleural effusion in this group was higher than that in other patient groups. The results for CS patients with and without osteotomy were compared, and the difference was not significant. Sixteen patients with pleural effusion underwent osteotomy, and 5 developed a pleural tear during the procedure. These findings were consistent with the results of our multivariate logistic regression analysis. If osteotomy procedure is considered, protecting the pleura intact is important to avoid this complication, especially in the convex side.

Spine surgeons are not highly familiar with the anterior approach for spine surgery, thus, this approach has a higher complication rate than the posterior approach[9,33,35]. Weis et al reported a 26% rate of minor complications in 98 idiopathic scoliosis patients who underwent anterior spinal fusion surgeries[35]. Grossfeld et al analyzed 599 patients who underwent anterior spinal fusion surgeries and found a 33% rate of minor complications[33]. Because the anterior approach involves the manipulation of the pleura, some authors have reported that post-operative hemothorax was associated with anterior procedures[33,35]. When instrumented video-assisted thoracoscopic surgery(VATS) is used for scoliosis treatment, the pleura are usually not sutured, thus increasing the risk of pleural effusion[25,36,37]. We had compared the occurrence of the patients who underwent anterior only surgery with those who underwent posterior only surgery, the result showed that the difference was relatively significant(P = 0.046). In the Grossfeld et al’s report, pleural effusion was identified in 21 patients (3.5%,21/599)[33]. In Nakai et al’s series, among the 6 patients with pleural effusion, 4 underwent anterior spinal fusion[9]. Newton et al reported 112 cases of video-assisted thoracoscopic anterior spinal release and fusion, and 66(59%) cases developed pleural effusion, including one case of chylothorax[36]. Among our 11 patients treated with VATS instrumentation correction surgery, 1 patient developed pleural effusion, and 1 developed chylothorax, as reported in our previous study[25].

In summary, pleural effusion occurred at a rate of 0.84% (28/3325) in spinal deformity surgeries. Hemothorax was the most common type of pleural effusion, and chylothorax and subarachnoid-pleural fistula were rarer. Adult patients(≥18 years old), congenital scoliosis, osteotomy and thoracoplasty were risk factors for pleural effusion. In these conditions, the spine surgeons should keep in mind to leave the pleura intact to avoid this complication. However, most patients with this complication could be treated conservatively with a good prognosis.

## Abbreviations

A: anterior
AIS: adolescent idiopathic scoliosis
ASF: anterior spinal fusion
A+P: anterior+posterior
CS: congenital scoliosis
HV: hemivertebra
IS: idiopathic scoliosis
NF: neurofibromatosis scoliosis
NMS: neuromuscular scoliosis
P: posterior
PSO: pedicle subtraction osteotomy
SPO: Smith-Peterson osteotomy
VCR: vertebral column resection
